# Editorial: Neuroendocrine Research in Health and Disease

**DOI:** 10.3389/fnins.2020.00176

**Published:** 2020-03-24

**Authors:** Yu-Feng Wang, Xuequn Chen, Lei Sha, Keith M. Kendrick, Leo T. O. Lee, Christopher H. K. Cheng

**Affiliations:** ^1^Department of Physiology, Harbin Medical University, Harbin, China; ^2^Division of Neurobiology and Physiology, Department of Basic Medical Sciences, School of Medicine, Zhejiang University, Hangzhou, China; ^3^Department of Neuroendocrine Pharmacology, School of Pharmacy, China Medical University, Shenyang, China; ^4^Key Laboratory for Neuroinformation, The Clinical Hospital of Chengdu Brain Science Institute, University of Electronic Science and Technology of China, Chengdu, China; ^5^Faculty of Health Sciences, University of Macau, Shanghai, China; ^6^School of Biomedical Sciences, The Chinese University of Hong Kong, Hong Kong, China

**Keywords:** hormone, hypothalamus, innervation, neuropeptide, receptor

To facilitate interactions between Chinese neuroendocrine researchers and the international neuroendocrine community, promote the growth of neuroendocrine research, and explore optimal treatment strategies for neuroendocrine diseases, we opened this topic on February 10, 2018. At the end of this topic, we had reviewed 31 manuscripts and accepted 22 for publication in the Neuroendocrine Science Section of the Journals of Frontiers in Neuroscience and Frontiers in Endocrinology. Among these papers, there were 19 original research contributions and three reviews. The research reported in these papers covered neuroendocrine regulation of mental activity, novel neuroendocrine pathways, peripheral effects of neuropeptides, receptor signaling, neuroendocrine diseases and treatments, and other associated areas.

## Neuroendocrine Regulation of Mental Activity

Kendrick and colleagues initiated the publications by presenting findings that stated “oxytocin (OT) facilitates empathic- and self-embarrassment ratings through attenuating amygdala and anterior insula responses” (Geng et al.). OT, a hypothalamic neuropeptide, can enhance emotional empathy in association with reduced amygdala activation. By using a randomized, double-blind placebo controlled functional MRI experiment on 70 male and female subjects, the authors investigated the effects of intranasal OT (40 IU) on behavioral and neural responses to embarrassment experienced by the self or others. The results demonstrated that OT increases ratings of self- and other embarrassment and that this is associated with reduced physiological arousal and activity in neural circuitry involved in emotional arousal. Interestingly, the neural effects of OT are stronger in individuals with high trait anxiety, suggesting that they may particularly reduce their anxiety in embarrassing situations.

Next, Lin et al. reported that high plasma levels of irisin, a humoral factor important for metabolism and homeostasis of energy balance, are associated with early cognitive deficits in Chinese patients with Type 2 diabetes mellitus (T2DM). In this study on 133 Chinese patients with T2DM, the authors found that a higher level of irisin in the plasma is associated with impaired overall cognition, specifically in executive function. It is likely that irisin functions both as “a sports pill” and as a signal of impaired cognition.

In a morphometric MRI study, Yang H. et al. reported alterations in cortical thickness in young male patients with childhood-onset adult growth hormone deficiency. They revealed a close correlation between serum growth hormone (GH)/insulin-like growth factor-1 (IGF-1) levels and age-related cognitive function. This correlation is based on alterations in cortical thickness in different brain lobes/regions.

In another study, Liu X. Y. et al. reported that intranasal OT alleviated maternal postpartum depression while improving milk production. Findings demonstrated that pup deprivation can evoke aberrant maternal behavior and hypogalactia in rat dams and that OT significantly improves these symptoms. In the supraoptic nucleus (SON), a major source of brain OT, intranasal administration can reverse pup deprivation -evoked reduction of c-Fos and increases glial fibrillary acidic protein (GFAP) filaments. Notably, OT also increases plasma levels of adrenocorticotropic hormone and corticosterone in pup-deprived dams. These Liu X. Y. et al. findings highlight the therapeutic potential of OT in pup-deprived dams by restoring the activity of the hypothalamic OT-secreting system involving modulation of astrocytic plasticity. However, OT's activation of the hypothalamic pituitary adrenal (HPA) axis likely compromises the facilitatory effects of intranasal administration.

Involvement of the HPA axis in mental activity has been well-known for decades. In the report by Zhou et al., the causal association between cortisol levels and depression is verified through a Mendelian randomization approach in a CORNET consortium of 12,597 participants (Zhou and Qiao). The result confirmed that a genetic predisposition to higher serum morning cortisol levels is associated with an increased depression score. Moreover, Wu R. et al. reported that prenatal dexamethasone exposure causes depression-like behavior in adult rats due to mitochondrial dysfunction. Intervention with treadmill exercise in early life can reverse this depression through improving mitochondrial function in the hippocampus (Wu T. et al.). Consistent with these latter findings, Qin et al. show that in rhesus macaques, a non-human primate, chronic glucocorticoid exposure leads to depression-like behavior and reduces dopamine (DA) levels in cerebrospinal fluid (CSF). Paradoxically, this treatment decreases cortisol concentration in the blood but increases them in hair. Thus, chronic glucocorticoid exposure may disturb both acute and chronic HPA axis reactivity, which eventually disturbs neurotransmitter systems and leads to a depression-like phenotype.

In addition to OT and corticosteroid hormones, sex steroid hormones are also involved in the regulation of mental activity. A paper by Cheng et al. demonstrated that androgens and androgenic signaling are involved in the occurrence of depression and anxiety. Following exposure to androgen during pregnancy, rats manifest depressive, anxious, and stereotypical behaviors in the adolescent period. These phenotypes are possibly associated with changes in neurogenesis in the dentate gyrus of the hippocampus.

## Novel Neuroendocrine Pathways

Neuroendocrine regulation of bodily activity can be influenced by direct neural innervation along with the actions of circulating neurohormones. In studying the mechanism underlying gastroparesis in patients with Parkinson's disease (PD), Yang Y.-L. et al. demonstrated that DA neurons in the substantia nigra (SN) project to and activate the lateral hypothalamic nucleus (LH) via the D1 receptor. LH neurons expressing orexin A also innervate the dorsal motor nucleus of the vagus (DMV). By activating the orexin receptor 1 of DMV, orexin A from the LH can alter gastric motility. Reduction of DA in the SN of PD patients decreases the activity of this SN-LH-DMV pathway, which ultimately leads to gastric dysfunction via the vagal nerve (Yang Y.-L. et al.).

Zhou et al. also reported that dopaminergic neurons in the SN are involved in the regulation of glucose metabolism through corticotropin-releasing hormone (CRH) neurons that express DA receptor 2 (D2) in the hypothalamic paraventricular nucleus (PVN). Bilateral SN lesions decrease glucose tolerance in rats by down-regulating the D2 receptor and up-regulating CRH in the PVN (Zhou et al.). These findings highlight that high prevalence of glucose metabolism abnormalities in PD patients associates with down-regulation of D2 and up-regulation of CRH in the PVN.

The review by Li et al. systematically demonstrated how pancreatic endocrine secretion is under the regulation of extrapancreatic nerves projecting to the islet directly, or converging on intrapancreatic ganglia, innervated by sympathetic, parasympathetic, enteric, sensory nerve fibers, and other intrapancreatic ganglia. It also highlighted the necessity of clarifying the roles and the mechanisms of intrapancreatic ganglia in physiological and disease states of the pancreas (Li et al.).

## Peripheral Effects of Neuropeptides

Neuropeptides have extensive influences on bodily functions and this view is illustrated further in the current topic. Zhang J. et al. or demonstrated that the neuropeptide substance P released from primary sensory fibers promotes proliferation of adult pancreatic ductal cells—one of the important sources of pancreatic islet β-cell neogenesis—but not their differentiation into β-cells. The observed effect of substance P involves the NK-1 receptor and Wnt signaling pathway. This finding indicates that lack of substance P may be a possible risk factor for diabetes development (Zhang N. et al.).

The review by Wang et al., comprehensively demonstrated the therapeutic potential of OT in atherosclerotic cardiovascular disease and the underlying mechanisms and signaling pathways. This review first linked atherosclerosis with varieties of immunometabolic disorders that can suppress OT receptor (OTR) signaling in the cardiovascular system (CVS). Next, the authors discussed evidence for this and the cellular and molecular mechanisms underlying CVS protective effects of OT. Finally, the authors also presented evidence for OT-evoked cardiovascular disturbance and the strategy for applying OT safely in clinical practice.

## Receptor Signaling

Different hormones can have multiple receptors and the effects of activating a specific receptor rely on specific signaling events. The report by Ding et al. showed that activation of the G protein-coupled estrogen receptor (GPER) elicits the release from calcium stores and phosphorylation of the mu-opioid receptor by regulating reproduction and metabolism in human neuroblastoma cells. Activation of GPER is followed by rapid calcium mobilization, translocation, and activation of protein kinase C-α and -ε at the plasma membrane leading to mu-opioid receptor phosphorylation. The GPER-mediated rapid calcium signal may also transmit to the nucleus to impact on gene transcription (Ding et al.). Such a signaling cascade may play an important role in the regulation of opioid signaling in the brain.

The review by Wang et al., discussed cellular and molecular mechanisms underlying cardiovascular protective effects of OT. The OT receptor signals mainly belong to the reperfusion injury salvage kinase pathway composed of PI3K-Akt-endothelial nitric oxide synthase cascades and extracellular signal-regulated protein kinase 1/2 (ERK 1/2). Additionally, AMP-activated protein kinase, Ca^2+^/calmodulin-dependent protein kinase signaling, and many others can influence OTR signaling related to cardiovascular protection. These signaling events coordinate at many levels to suppress metabolic disorders, reduce the production of inflammatory cytokines, and inhibit the activation of apoptotic pathways, particularly endoplasmic reticulum stress and mitochondrial oxidative stress. This comprehensive review expands our understanding of the immunoregulatory functions of OT.

## Disease and Treatment

Neuroendocrine studies have established the involvement and treatment effects of neuropeptides in a variety of diseases. In their study, Ma et al. found for the first time that the expression of OT and OTR declines in human colorectal tissues as malignancy of colorectal cancer (CRC) increases. They reported that OT can suppress the expression of tumor-associated immunosuppressive proteins fibroblast activation protein-α (FAPα) and chemokine (C-C motif) ligand 2 (CCL-2). The reduction in OT-OTR expression can unleash the expression of FAPα and CCL-2 and, thus, facilitates CRC metastasis. Importantly, OT can reduce the invasion ability of human CRC cells. Although the authors limited their findings to colorectal adenocarcinoma, the therapeutic potential of directly applying OT to inhibit CRC migration is worthy of further exploration.

The report by Zhu et al., studied the spatial distribution patterns of primary age-related tauopathy (PART) in subcortical nuclei, including the neuroendocrine hypothalamus of post-mortem human brains. They found that the prevalence and severity of tau pathology in subcortical nuclei of PART and AD positively correlate with the stage of NFT Braak neurofibrillary tangles, suggesting that these nuclei are increasingly involved as PART and AD progress. Thus, these subcortical nuclei are probably the sites initially affected by aging associated tau pathology, especially brainstem nuclei.

Zou et al., reviewed the role of leptin in mood disorders and neurodegenerative diseases and summarized findings that leptin can improve learning and memory, affect hippocampal synaptic plasticity, exert neuroprotective efficacy, and reduce the risk of several neuropsychiatric diseases, including depression. These effects are associated with leptin regulation of the release of neurotransmitters and neurotrophic factors and can reverse dysfunction in the HPA axis.

Sheng et al. demonstrated an involvement of mitogenic signaling in the regulation of thyroid proliferation by insulin glargine and human insulin. Their results show that therapeutic doses of glargine do not stimulate thyroid cell proliferation, despite longer-lasting hypoglycemic control than human insulin. However, high doses of these insulin products can stimulate thyroid cell proliferation, which should therefore be a consideration in the clinical use of insulin glargine.

## Others

The topic also includes neuroendocrine studies in other associated research areas. Liu L. L. et al. reported CSF metabolite profiles in neurosyphilis patients following an untargeted metabolomics analysis. These differential metabolites may potentially improve neurosyphilis diagnostics in the future and deserve further exploration. Xiao et al. demonstrated that prenatal ethanol exposure produces age-dependent changes in glucose metabolism, pancreatic morphology, and function in male offspring rats. This intrauterine programming alteration in the GC-IGF1 axis may contribute to prenatal and postnatal pancreatic dysplasia and impaired insulin biosynthesis in male offspring.

In addition, Wu R. et al. presented research suggesting that brief mindfulness meditation can significantly improve emotion processing. The beneficial effect correlates with reduced activity of the HPA-axis and stress responses (Wu R. et al.). The report by Zhang J. et al. revealed that voluntary wheel running reverses deficits in social behavior following chronic social defeat stress in mice due to the reversal of reduced levels of tyrosine hydroxylase in the ventral tegmental area and the D2 receptor in the nucleus accumbens shell.

Taken together, our topic submissions demonstrate that Chinese neuroendocrine researchers are contributing significantly to the field of neuroendocrinology. While many challenges remain, the growth of a new generation of Chinese neuroendocrine researchers will, we hope, result in even stronger contributions to novel approaches, concepts, and principles in both fundamental and clinical neuroendocrine research.

## Author Contributions

All six authors contributed to organizing the Research Topic, in which XC has taken the most editorial assignments. Y-FW wrote the first draft, all participated in the correction of this editorial and KK made the final revision.

### Conflict of Interest

The authors declare that the research was conducted in the absence of any commercial or financial relationships that could be construed as a potential conflict of interest.

